# 08. Concomitant Administration of the Adjuvanted Recombinant Zoster Vaccine (RZV) with 13-Valent Pneumococcal Conjugate Vaccine (PCV13) Is Safe and Does Not Interfere with Immunogenicity of Either Vaccine in Adults Aged ≥ 50 Years

**DOI:** 10.1093/ofid/ofab466.211

**Published:** 2021-12-04

**Authors:** Ji-Young Min, Agnes Mwakingwe-Omar, Megan Riley, Lifeter Yenwo Molo, Jyoti Soni, Ginette Girard, Jasur Danier

**Affiliations:** 1 GSK, Rockville, MD, USA, Rockville, Maryland; 2 GSK, Rockville, Maryland; 3 GSK, Wavre, Belgium, Wavre, Brabant Wallon, Belgium; 4 GSK, Bangalore, India, Bangalore, Karnataka, India; 5 Diex Recherche Inc. Sherbrooke, Sherbrooke, QC, Canada, Sherbrooke, Quebec, Canada

## Abstract

**Background:**

This study assessed non-inferiority of humoral immunogenicity, reactogenicity, and safety of RZV when the 1st dose was co-administered with PCV13 in adults ≥ 50 years of age (YOA) compared to sequential administration.

**Methods:**

In this phase 3b, open-label, multi-center study (NCT03439657), adults were randomized 1:1 to receive either the 1st RZV dose co-administered with PCV13 at day (D)1 and the 2nd RZV dose at month (M)2 (Co-Ad group), or PCV13 at D1, the 1st RZV dose at M2 and the 2nd RZV dose at M4 (Control group). Co-primary confirmatory objectives were: (i) vaccine response rate (VRR) to RZV at 1 month post-dose 2 in Co-Ad group; (ii) non-inferiority of humoral responses to RZV (1 month post-RZV dose 2) and PCV13 (1 month post-PCV13) in Co-Ad group compared to Control group. Solicited adverse events (AEs) until D7 post-vaccination and unsolicited AEs until D30 post-vaccination were recorded. Serious AEs (SAEs) and potential immune-mediated diseases (pIMDs) were collected through 12 months post-RZV dose 2. Immunogenicity was performed in the per-protocol set (PPS) and safety analyses in the exposed set.

**Results:**

Of 912 vaccinated adults, 863 were included in PPS (Co-Ad: 427; Control: 436). VRR for anti-glycoprotein E antibody concentrations was 99.1% in Co-Ad group. The predefined non-inferiority criteria for the humoral immune responses to RZV and PCV13 were met (Table 1). The overall frequency of solicited local AEs after RZV and PCV13 was comparable between Co-Ad and Control groups. Pain was the most common solicited local AE (Figure 1). The frequency of solicited general AEs was similar for the 1st RZV dose when co-administered with PCV13 or alone (57.4% vs 54.6%). Myalgia and fatigue were the most common solicited general AEs (Figure 2). The frequency (Co-Ad: 21.2%; Control: 23.1%) and nature of unsolicited AEs were balanced between groups. None of the reported SAEs, fatal SAEs, or pIMDs were vaccine-related.

Table 1. Co-primary confirmatory objectives: vaccine response rate (VRR), and non-inferiority of the immune responses to RZV (1 month post-dose 2) and to PCV13 (1 month post-vaccination) in the Co-Ad group vs the Control group (per-protocol set)

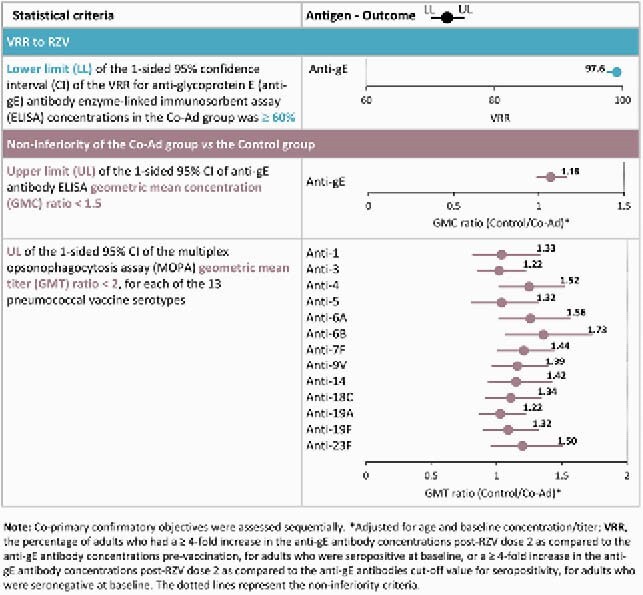

Figure 1. The incidence of solicited local adverse events (AEs) occurring within 7 days post-vaccination (overall/adult, exposed set)

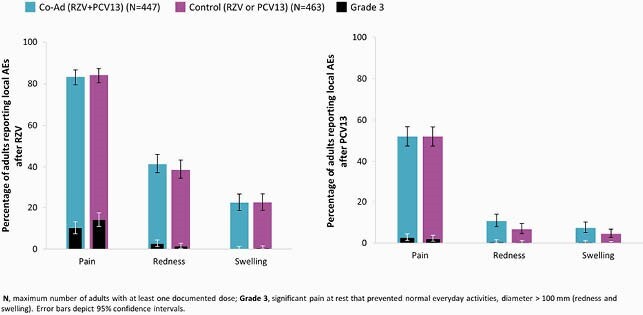

Figure 2. The incidence of solicited general adverse events (AEs) post-dose 1 occurring within 7 days post-vaccination (exposed set)

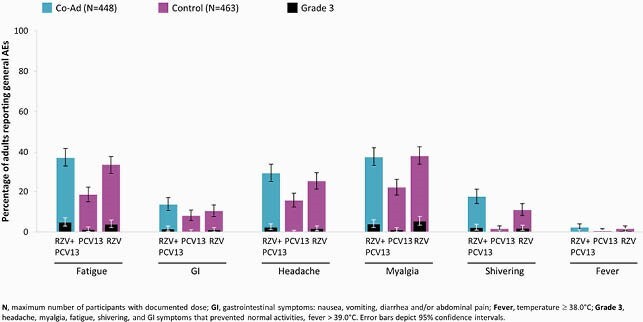

**Conclusion:**

Co-administration of the 1st RZV dose with PCV13 showed non-inferior immune responses to sequential administration. The reactogenicity and safety of RZV in the Co-Ad group were within the range of the established safety profile of RZV. Co-administration of RZV with PCV13 may improve vaccination rates in ≥ 50 YOA population.

**Funding:**

GlaxoSmithKline Biologicals SA

**Disclosures:**

**Ji-Young Min, PhD**, **GSK group of companies** (Employee) **Agnes Mwakingwe-Omar, MD, PhD**, **GSK group of companies** (Employee) **Megan Riley, PhD**, **GSK group of companies** (Employee, Shareholder) **Lifeter Yenwo Molo, BsC(hons) MSc(hons**), **GSK group of companies** (Employee) **Jyoti Soni, MA**, **GSK group of companies** (Employee) **Ginette Girard, MD**, **Diex Recherche Inc. Sherbrooke** (Scientific Research Study Investigator) **Jasur Danier, MD**, **GSK group of companies** (Employee, Shareholder)

